# Nutcracker Cuboid Fracture: A Case Report and Review

**DOI:** 10.1155/2018/3804642

**Published:** 2018-04-03

**Authors:** Alan Lucerna, James Espinosa, Nicholas Butler, Ashley Wenke, Nicole Caltabiano

**Affiliations:** ^1^Department of Emergency Medicine, Rowan University SOM and Jefferson Health, Stratford, NJ, USA; ^2^Department of Podiatric Medicine, Jefferson Health, Stratford, NJ, USA; ^3^Rowan University SOM, Stratford, NJ, USA

## Abstract

Here we report the case of a 20-year-old female restrained driver who presented to the emergency department (ED) after a motor vehicle accident. She sustained an isolated fracture of her left cuboid, consistent with a nutcracker cuboid fracture. A cuboid fracture is considered rare. It is even more uncommon for a cuboid fracture to occur in isolation, without other associated injuries to the foot. We discuss the mechanism, relevant anatomy, diagnosis, and principles of treatment of the nutcracker cuboid fracture.

## 1. Introduction

The anatomy of the cuboid is complex, with six articular surfaces involvement in all of the intrinsic movements of the midfoot and hindfoot [1]. Due to this anatomy, a cuboid fracture is considered rare. It is even more uncommon for a cuboid fracture to occur in isolation, without other associated injuries to the foot [2]. We report the case of a 20-year-old female restrained driver who presented to the emergency department (ED) after a motor vehicle accident.

## 2. Case Report

**Figure 1:**
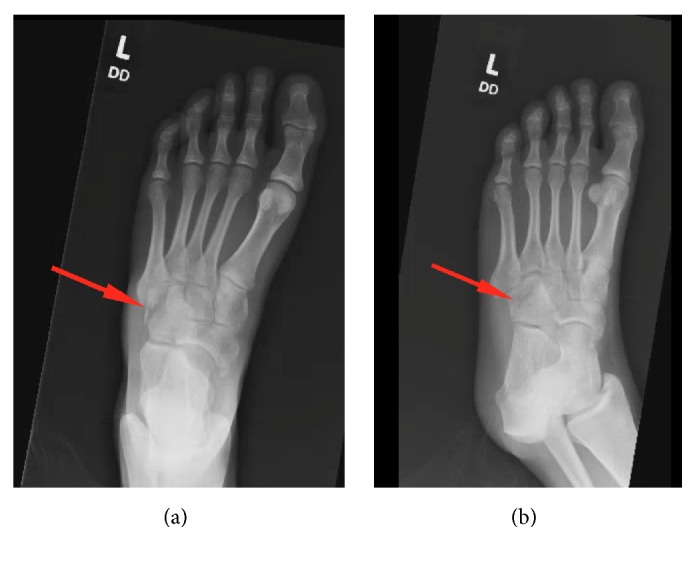
An X-ray of the left foot showed a commuted fracture of the cuboid bone with mildly displaced fragments, as well as moderate soft tissue edema (red arrows).

A 20-year-old female restrained driver presented to the emergency department (ED) after motor vehicle accident. She complained of left clavicular and left foot pain. A left clavicular fracture was found on exam and on the imaging studies. An X-ray of the left foot showed a commuted fracture of the cuboid bone with mildly displaced fragments, as well as moderate soft tissue edema (Figure 1). Clinical exam did not show evidence of other foot or leg injuries. The rest of her exam was unremarkable. An orthopedic consult was obtained in the emergency department. A posterior fiberglass splint was recommended as well as non-weight bearing on the affected foot. The patient was to follow up on an outpatient basis with orthopedic surgery. The fracture was consistent with a nutcracker cuboid fracture.

## 3. Discussion

### 3.1. Incidence of Cuboid Fractures

A cuboid fracture is considered rare. It is even more uncommon for a cuboid fracture to occur in isolation, without other associated injuries to the foot [2]. This is said to be because of a relatively protected position in the midfoot [3].

### 3.2. Classification

According to Smith et al., who performed a large retrospective study of cuboid fractures, there is no current widely accepted classification for cuboid fractures and there are no long-term outcome studies [3].

### 3.3. Anatomy

The anatomy of the cuboid is complex, with six articular surfaces' involvement in all of the intrinsic movements of the midfoot and hindfoot [1]. The cuboid constitutes part of the lateral longitudinal arch of the foot [4]. It is the only bony structural support for the lateral column of the midfoot [5]. Of note, the tendon of the peroneus longus courses in a groove on the inferior surface of the cuboid bone [4]. The plantar surface of the cuboid has attachments for the short and long plantar ligaments [2]. Because of these important anatomic elements, “any disturbance of the articular surfaces of the cuboid can lead to a profound disruption of the movement and biomechanics of the midfoot” [2]. Loss of length of the lateral column due to a cuboid fracture can result in abduction of the forefoot with a planus deformity. There can be compensatory eversion of the hindfoot [2].

### 3.4. Nutcracker Mechanism and Fracture

Isolated fractures of the cuboid, as in the case presented, are rare. It is more common to find cuboid fractures from a crush injury, in association with other foot fractures and injuries [1, 3, 4]. Hermel and Gershon-Cohen described a mechanism that can result in an isolated cuboid fracture, in which forced abduction of the forefoot, generally in combination with an axial load, can compress the cuboid between the bases of the 4th and 5th metatarsals and the calcaneus [6]. Plantar flexion of the foot may be involved at the time of injury (Gaines). The authors likened this compression to a nutcracker effect and noted that this mechanism can result in a “nutcracker cuboid fracture” [3, 6–8]. It is likely that the patient described had just such a force (axial load with abduction) from the motor vehicle accident. Nutcracker cuboid fractures have been described in ballet dancers, likely due to repetitive axial loading of the foot [9]. Children have been described with nutcracker cuboid injuries associated with horseback riding [10]. Hsu et al. note that “little has been published on the frequency, diagnosis or treatment of the nutcracker fracture in the pediatric population.” Nutcracker cuboid injuries in children can be difficult to diagnose [11].

### 3.5. Diagnosis

The diagnosis may be difficult to make and can be missed on the initial evaluation [5].

Imaging includes X-rays of the foot. An oblique view may help to define the calcaneal-cuboid and metatarsal-cuboid relationship [7]. Computerized tomography (CT) imaging may be helpful when the diagnosis is unclear [5].

### 3.6. Treatment

Little high-quality evidence can be found on the best treatment for navicular-cuboid fractures. The treatments described in the literature include such options as immobilization with casting, external fixation, and open reduction internal fixation with or without bone grafting. Midtarsal fusion has been described. Yu et al. note that “the indications and the best method of surgical treatment have not been established due the rarity of the fracture and the paucity of literature” [5].

Koch makes a similar point. “Due to the limited number of reported cases of nutcracker fractures, the best method of treatment has not been determined” [12]. Ohmori et al. reported a “new treatment plan” for an isolated nutcracker injury—arthroscopic elevation of depressed bone fragments and the use of a bone biopsy needle to fill in the large defect with artificial bone [13]. Nondisplaced fractures may be treated with a short leg cast and non-weight-bearing status [14]. Comminuted fractures will more likely need an operative procedure [14]. Smith et al. note that when a nutcracker cuboid fracture is associated with lateral column shortening, lengthening of the lateral column, open reduction internal fixation (ORIF), and bone grafting may be needed [3]. External fixation may have a role in some cases [8]. Yu et al. recommend open treatment for cuboid fractures when there is a one millimeter or more shortening of the lateral column [5].

### 3.7. Outcomes

As noted by Fenton et al., “there are no long term reports of outcomes following fractures of the cuboid” [2]. The major concerns with outcome include pain, loss of the lateral structural integrity of the foot, and decreased range of motion of the lateral tarsometatarsal joints [5].

## 4. Conclusions

Cuboid fractures are considered rare, even more so without other associated injuries to the foot. It is likely that the patient described had just such a force (axial load with abduction) from the motor vehicle accident. Little high-quality evidence can be found on the best treatment for navicular-cuboid fractures.

Nondisplaced fractures may be treated with a short leg cast and non-weight-bearing status. Comminuted fractures will more likely need an operative procedure like open reduction internal fixation (ORIF) and bone grafting as well as placement of external fixators. There are no long-term reports of outcomes following fractures of the cuboid. The major concerns with outcome include pain, loss of the lateral structural integrity of the foot, and decreased range of motion of the lateral tarsometatarsal joints.
